# Supplementary material for: Fungal Pathogens Affecting the Production and Quality of Medical Cannabis in Israel
Source: Plants (Basel). 2020 Jul 13;9(7):882. doi: 10.3390/plants9070882 (PMC7412049; doi:10.3390/plants9070882)
Supplement: Supplementary file 1 [file plants-09-00882-s001.pdf]

Supplementary Table S1. Fungal isolates sampled from different plant parts in this study and identified to species by molecular methods.

Isolate Designation	Fungal Species Identified	Infected Plant Tissue	Isolation Season[a]	Primers[b]			
				ITS 4-5	EF1	RPB2	IGS
1A	*Fusarium solani*	Stem	Summer	+	+	-	-
2A	*Fusarium solani*	Stem	Summer	+	+	-	-
3A	*Fusarium keratoplasticum*	Stem	Summer	+	+	-	-
4A	*Fusarium keratoplasticum*	Stem	Summer	+	+	-	-
5A	*Fusarium petroliphilum*	Stem	Summer	+	+	-	-
6A	*Fusarium oxysporum*	Stem	Summer	+	+	-	-
7A	*Fusarium oxysporum*	Stem	Summer	+	+	-	-
10A	*Fusarium equiseti*	Inflorescence	Autumn	+	+	-	-
16A	*Trichothecium roseum*	Stem	Autumn	+	-	-	-
17A	*Trichothecium roseum*	Inflorescence	Autumn	+	-	-	-
19A	*Lasiodiplodia theobromae*	Root	Autumn	+	-	-	-
20A	*Lasiodiplodia theobromae*	Stem	Autumn	+	-	-	-
21A	*Lasiodiplodia theobromae*	Root	Autumn	+	-	-	-
22A	*Alternaria alternata*	Root	Autumn	+	+	+	-
23A	*Alternaria alternata*	Inflorescence	Autumn	+	+	+	-
24A	*Alternaria alternata*	Inflorescence	Autumn	+	+	+	-
25A	*Penicillium citrinum*	Inflorescence	Autumn	+	-	-	-
26A	*Cladosporium* sp.	Inflorescence	Autumn	+	-	-	-
27A	*Alternaria botrytis*	Inflorescence	Winter	+	+	+	-
28A	*Alternaria chlamydospora*	Inflorescence	Winter	+	+	+	-

29B	*Chaetomium globosum*	Leaves	Autumn	+	-	-	-
29C	*Alternaria alternata*	Leaves	Autumn	+	+	+	-
30A	*Alternaria alternata*	Inflorescence	Autumn	+	+	+	-
31A	*Alternaria* sp.	Leaves	Autumn	+	+	+	-
31B	*Alternaria alternata*	Leaves	Autumn	+	+	+	-
32A	*Cladosporium* sp.	Inflorescence	Autumn	+	-	-	-
33A	*Alternaria* sp.	Inflorescence	Autumn	+	+	+	-
33B	*Penicillium steckii*	Inflorescence	Autumn	+	-	-	-
34B	*Aspergillus flavus*	Leaves	Autumn	+	-	-	-
35A	*Cladosporium halotolerans*	Leaves	Autumn	+	-	-	-
35B	*Acremonium* sp.	Leaves	Autumn	+	-	-	-
36A	*Alternaria alternata*	Leaves	Autumn	+	+	+	-
36B	*Cladosporium cladosporioides*	Leaves	Autumn	+	-	-	-
37Ass2	*Alternaria* sp.	Leaves	Autumn	+	+	+	-
38B	*Alternaria* sp.	Leaves	Autumn	+	+	+	-
39A	*Sordaria* sp.	Leaves	Autumn	+	-	-	-
39B	*Alternaria alternata*	Leaves	Autumn	+	+	+	-
42A	*Cladosporium sphaerospermum*	Leaves	Autumn	+	-	-	-
43A	*Alternaria* sp.	Leaves	Autumn	+	+	+	-
44A	*Trichoderma harzianum*	Stem	Autumn	+	-	-	-
45A	*Trichoderma hamatum*	Root	Autumn	+	-	-	-
45Aparp	*Trichoderma hamatum*	Root	Autumn	+	-	-	-
46A	*Cladosporium* sp.	Root	Autumn	+	-	-	-
47A	*Alternaria alternata*	Leaves	Autumn	+	+	+	-
48A	*Trichoderma harzianum*	Root	Autumn	+	-	-	-
49A	*Aspergillus flavus*	Inflorescence	Autumn	+	-	-	-

49B	*Trichoderma harzianum*	Inflorescence	Autumn	+	-	-	-
53A	*Aspergillus niger*	Inflorescence	Winter	+	-	-	-
54A	*Botrytis cinerea*	Inflorescence	Spring	+	-	-	-
55A	*Alternaria alternata*	Leaves	Spring	+	+	+	-
56A	*Botrytis cinerea*	Inflorescence	Spring	+	-	-	-
57A	*Sclerotinia sclerotiorum*	Inflorescence	Spring	+	-	-	-
59A	*Stemphylium vesicarium*	Root	Spring	+	-	-	-
59B	*Alternaria alternata*	Root	Spring	+	+	+	-
59C	*Trichoderma citrinoviride*	Root	Spring	+	-	-	-
61A	*Alternaria* sp.	Inflorescence	Spring	+	+	+	-
62A	*Fusarium oxysporum*	Inflorescence	Spring	+	+	-	+
63A	*Botrytis cinerea*	Inflorescence	Spring	+	-	-	-
63B	*Stemphylium vesicarium*	Inflorescence	Spring	+	-	-	-
64A	*Fusarium oxysporum*	Inflorescence	Spring	+	+	-	-
64B	*Fusarium oxysporum*	Inflorescence	Spring	+	+	-	+
65A	*Botrytis cinerea*	Inflorescence	Spring	+	-	-	-
68-1A	*Alternaria alternata*	Inflorescence	Spring	+	+	+	-
68A	*Alternaria alternata*	Inflorescence	Summer	+	+	+	-
69-2A	*Alternaria alternata*	Inflorescence	Summer	+	+	+	-
70A	*Fusarium oxysporum*	Stem	Summer	+	+	-	-
71B	*Aspergillus westerdijkiae/ ochraceus*	Inflorescence	Summer	+	+	-	-
73B	*Aspergillus fumigatus*	Inflorescence	Summer	+	-	-	-
75A	*Fusarium oxysporum*	Inflorescence	Summer	+	+	-	-
77A	*Alternaria* sp.	Inflorescence	Summer	+	+	+	-
84A	*Alternaria alternata*	Leaves	Summer	+	+	+	-

85A	*Fusarium solani*	Stem	Summer	+	+	-	-
86B	*Fusarium verticillioides*	Stem	Summer	+	+	-	-
99A	*Fusarium proliferatum*	Stem	Summer	+	+	-	-
106A	*Aspergillus fumigatus*	Inflorescence	Autumn	+	-	-	-
106B	*Fusarium equiseti*	Inflorescence	Autumn	+	+	-	-
107C	*Alternaria alternata*	Inflorescence	Autumn	+	+	+	-
108B	*Fusarium oxysporum*	Inflorescence	Autumn	+	+	-	-
109A-1	*Fusarium solani*	Stem	Autumn	+	+	-	-
109A-2	*Fusarium solani*	Stem	Autumn	+	+	-	-
109A-3	*Fusarium solani*	Stem	Autumn	+	+	-	-
109A-4	*Fusarium falciforme*	Stem	Autumn	+	+	-	-
110A	*Penicillium olsonii*	Inflorescence	Autumn	+	-	-	-
111A	*Botrytis cinerea*	Inflorescence	Autumn	+	-	-	-
112A	*Fusarium brachygibbosum*	Inflorescence	Autumn	+	-	-	-
114A	*Fusarium brachygibbosum*	Inflorescence	Autumn	+	-	-	-
116A	*Sclerotinia sclerotiorum*	Inflorescence	Winter	+	-	-	-
117A	*Alternaria alternata*	Leaves	Winter	+	+	+	-
118A	*Cladosporium cladosporioides*	Leaves	Winter	+	-	-	-
119B	*Cladosporium cladosporioides*	Leaves	Winter	+	-	-	-
119D	*Chaetomium madrasense*	Leaves	Winter	+	-	-	-
119E	*Petriella sordida*	Leaves	Winter	+	-	-	-
120A	*Petriella sordida*	Leaves	Winter	+	-	-	-
120B	*Petriella sordida*	Leaves	Winter	+	-	-	-
121A	*Arthrinium guizhouense*	Inflorescence	Spring	+	-	-	-

122A	*Petriella sordida*	Inflorescence	Spring	+	-	-	-
138A	*Aspergillus niger*	Inflorescence	Winter	+	-	-	-
139A	*Fusarium equiseti*	Inflorescence	Winter	+	+	-	-
140A	*Alternaria alternata*	Inflorescence	Winter	+	+	+	-
141A	*Penicillium citrinum*	Inflorescence	Winter	+	-	-	-
142A	*Aspergillus flavus*	Inflorescence	Winter	+	-	-	-

[a] Seasons were determined as follows, Summer (June-August), Autumn (September-November), Winter (December-February) and Spring (March-May). [b] + Tested with appropriate primers, - not tested.
